# Metabolomic and Gene Expression Profiles Exhibit Modular Genetic and Dietary Structure Linking Metabolic Syndrome Phenotypes in *Drosophila*

**DOI:** 10.1534/g3.115.023564

**Published:** 2015-11-03

**Authors:** Stephanie Williams, Kelly Dew-Budd, Kristen Davis, Julie Anderson, Ruth Bishop, Kenda Freeman, Dana Davis, Katherine Bray, Lauren Perkins, Joana Hubickey, Laura K. Reed

**Affiliations:** * Department of Genetics, North Carolina State University, Raleigh, North Carolina 27695; † Department of Biological Sciences, University of Alabama, Tuscaloosa, Alabama 35487

**Keywords:** metabolomics, genotype-by-environment interaction, metabolic syndrome, *Drosophila*, canalization

## Abstract

Genetic and environmental factors influence complex disease in humans, such as metabolic syndrome, and *Drosophila melanogaster* serves as an excellent model in which to test these factors experimentally. Here we explore the modularity of endophenotypes with an in-depth reanalysis of a previous study by Reed *et al*. (2014), where we raised 20 wild-type genetic lines of *Drosophila* larvae on four diets and measured gross phenotypes of body weight, total sugar, and total triglycerides, as well as the endophenotypes of metabolomic and whole-genome expression profiles. We then perform new gene expression experiments to test for conservation of phenotype-expression correlations across different diets and populations. We find that transcript levels correlated with gross phenotypes were enriched for puparial adhesion, metamorphosis, and central energy metabolism functions. The specific metabolites L-DOPA and *N*-arachidonoyl dopamine make physiological links between the gross phenotypes across diets, whereas leucine and isoleucine thus exhibit genotype-by-diet interactions. Between diets, we find low conservation of the endophenotypes that correlate with the gross phenotypes. Through the follow-up expression study, we found that transcript-trait correlations are well conserved across populations raised on a familiar diet, but on a novel diet, the transcript-trait correlations are no longer conserved. Thus, physiological canalization of metabolic phenotypes breaks down in a novel environment exposing cryptic variation. We cannot predict the physiological basis of disease in a perturbing environment from profiles observed in the ancestral environment. This study demonstrates that variation for disease traits within a population is acquired through a multitude of physiological mechanisms, some of which transcend genetic and environmental influences, and others that are specific to an individual’s genetic and environmental context.

Since the 1960s, the documented proportion of the United States population that is obese, which is defined by a body mass index exceeding 30 kg/m^2^, has increased by nearly 150%. Presently, 69% of Americans are overweight or obese (body mass index >25) (O’Rahilly and Farooqi 2006; Yamada *et al.* 2007; Monda *et al.* 2010; Ogden *et al.* 2014), and the percentage of obese individuals is expected to grow to more than 60% in many states by 2030 (http://www.rwjf.org/content/dam/farm/reports/reports/2012/rwjf401318). Moreover, the rates of the comorbidities associated with obesity, such as insulin resistance, elevated circulating blood lipids, and elevated blood pressure, have concomitantly increased. Collectively, these comorbidities are referred to as metabolic syndrome, or MetS, a syndrome that is contributing to a national epidemic of type 2 diabetes and cardiovascular disease.

Similar notable transitions to increased prevalence of MetS are actively occurring in many other countries. This drastic phenotypic transition can be attributed primarily to a shift toward a more Westernized environment, characterized by reduced physical activity and increased caloric intake. Yet, despite the obvious significant impact of lifestyle changes on public health, (Schulz *et al.* 2006; Musselman *et al.* 2011; Rulifson *et al.* 2002) there also remains a substantial component of genetic variation contributing to an individual’s risk of developing MetS and associated diseases (Yamada *et al.* 2007; Monda *et al.* 2010; O’Rahilly and Farooqi 2006). Disentangling the relative contributions of genetic and environmental factors to the mechanism of a complex human disease such as MetS is daunting because of the expense of procuring the enormous sample sizes necessary to make statistically valid conclusions, particularly in the face of tremendous genetic and lifestyle variation. However, it is becoming increasingly evident that we must employ some strategy to understand the mechanisms linking genes, the environment, and these correlated diseases. Fortunately, model organisms such as *Drosophila* can provide such a strategy.

Because of our shared evolutionary history, *Drosophila* and humans share many homologous physiological systems, including those relevant to the development of MetS, such as the insulin signaling pathway, central metabolism, innate immune function, and heart physiology (Reed *et al.* 2010, 2014; Musselman *et al.* 2011; Rulifson *et al.* 2002; Bodmer and Venkatesh 1998; Hoffmann and Reichhart 2002; Wessells *et al.* 2004). But unlike humans, *Drosophila* are a highly tractable experimental system and have been useful for a variety of systems biology-style experiments (Harbison *et al.* 2009; Chintapalli *et al.* 2013; Tennessen *et al.* 2014; Hoffman *et al.* 2014; Reed *et al.* 2014). In the laboratory, unlimited number of genetically identical *Drosophila* individuals can be exposed to different environmental conditions to test how a specific genotype reacts to changes in environment, thus allowing researchers to isolate the environmental effect on phenotype. Correspondingly, different genotypes of *Drosophila* can be measured in the same environment to understand how genetic variation contributes to phenotypic variation. Therefore, by using this multifactorial approach, it is possible to partition the genetic, environmental, and genotype-by-environment interaction effects determining the overall variation in a phenotype within a population.

Using this approach in previous studies, we observed highly significant genetic, dietary, and genotype-by-diet variation for each of the gross phenotypes of weight, total sugar, and triglycerides (Supporting Information, Figure S1) (Reed *et al.* 2010, 2014). Additionally, we consistently found that the genetic variance and the genotype-by-diet interaction effects account for a much larger proportion of the phenotypic variance than the diet effects alone for gross phenotypes and gene expression profiles (Figure S1) (Reed *et al.* 2010, 2014). However, the overall metabolite profiles showed a much stronger signature of dietary variance than the gross phenotypes or the gene transcription profiles (Reed *et al.* 2014).

Taken together, these results support the hypothesis that how an environmental or lifestyle transition will affect an individual largely depends on that individual’s genetic make-up rather than a generalized species-level physiological reaction. Thus, understanding the mechanisms driving the increasing incidence of MetS associated with Westernized-lifestyle and subsequently identifying methods to prevent and treat MetS requires us to carefully dissect the specific mechanisms of the genotype-by-environment interaction. Here we extend the analyses performed in Reed *et al.* (2014) to probe the variance profiles of the individual gene transcripts and metabolites and explore how they relate to the MetS-like phenotypes in *Drosophila melanogaster*. We identify multiple individual molecular signals (endophenotypes) that are strongly correlated with MetS and link together the gross phenotypes.

Through this work, we aimed to answer three primary questions: (1) How are genotype-by-diet interaction effects reflected in metabolomic and gene expression profiles, and do these profiles inform prediction of genotype-by-diet effects on MetS-like phenotypes? (2) Which endophenotypes link the gross phenotypes of body weight, triglyceride storage, and total sugar? (3) Which endophenotypes are predictive of gross phenotype in different dietary conditions? By integrating across these physiological levels and leveraging the added structure induced by genetic and dietary variation, we have identified several exciting new avenues of mechanistic exploration for understanding the causes of obesity and MetS.

## Materials and Methods

### Experimental design

#### Original population sample generation:

The initial portion of the data analyzed to a greater depth here has been reported previously in Reed *et al.* (2014), and the experimental design for sample generation is provided therein. To summarize in brief, an analysis was performed on 20 wild-type inbred genetic lines representing a diversity of dietary reaction norms for pupal weight and larval lipid storage originally collected from the wild populations in North Carolina and Maine. Reaction norms for each line and phenotype are shown in Figure S1. Four cornmeal-based diets that were identical except that they varied in their sugar and fat content were used to raise the larvae [the rationale for the diets has been described previously in detail (Reed *et al.* 2010, 2014)]. The diets were as follows: normal (4% sucrose by weight), control (0.75% glucose by weight), high sugar (4% glucose), and high fat (0.75% glucose, 3% added saturated fat—coconut oil). Food vials were seeded with 50 first instar larvae, which then developed either to third instar larvae or into pupae for phenotype measurement.

Larval samples were taken from pools of three to five vials of third instar after a 6-hr fasting period. TAG and total larval sugar content (glucose and trehalose) levels were determined on two samples of six larvae with the Sigma Triglyceride Determination and Sigma Glucose Assay kit, respectively, as described in Reed *et al.* (2010). Two samples each of six and 30 larvae were snap-frozen in liquid nitrogen for metabolomic and expression analyses, respectively. In addition, two food vials were allowed to develop to the pupal stage to measure pupal weight (individual wet weight of up to 15 mature male pupae) and were collected as described in Reed *et al.* (2010). Each genetic line was tested on all diet treatments simultaneously in randomized blocks of four synchronized lines per week and replicated at three independent time points (n = 240).

RNA samples for whole-genome expression profiling were extracted with MagMAX-96 for Microarrays Kit (Ambion; #AM1839) with the “Spin Procedure” and an additional TurboDNAse treatment (as described in the no-spin protocol). RNA quality and quantity were assessed with a Nanodrop spectrophotometer and by electrophoresis gel. Whole-genome expression analysis was performed with Nimblegen 12-plex microarrays by using Nimblegen protocols for cDNA synthesis and hybridization. Slides were imaged on a GenePix 5-micrometer scanner. Data were extracted from the image files with the Nimblescan software then transferred to JMP-Genomics for subsequent analyses. A total of 11,650 genes (of a possible 15,595) were expressed consistently at detectable levels (n = 219). All of the expression data from this study are deposited at the GEO database under accession number GSE50745.

Metabolomic profiling was performed by gas chromatography-mass spectrometry and is described in detail in Reed *et al.* (2014). To summarize in brief, daily randomized blocks of 15−22 unique samples were prepared and run on a Thermo Scientific DSQ II Series Single Quadrupole gas chromatography-mass spectrometry with an electron impact source and an Agilent DB-5 column run in splitless mode with a 30-minute temperature ramp (total n = 425). Dilution series of pooled standards were run at the beginning, middle, and end of each day to generate a standard curve, and PMIX standards were run at the beginning and end to allow for retention time calibration (Kovats retention index). Chromatograms were aligned and molecular targets were called with AnalyzerPro (http://www.spectralworks.com/analyzerpro.asp). After quality control filtering, 187 putative metabolites were proposed and their candidate compound ID was determined by searching their profiles against the publically available NIST database. Sixty of the metabolites were confirmed with chemical standards, and 124 were matched to chemical class (*e.g.*, amino acid or monosaccharide) on the basis of database comparisons, facilitated by ChemBioDraw 12.0 (http://www.cambridgesoft.com//) for visual compound ID and classification. Target/metabolite identities can be found in File S7.

#### Confirmation of gene expression patterns with diet:

To generate the new dataset used confirm expression patterns, 45 genetic lines raised on the control and high-fat dietary treatments were used for the follow-up experiment to confirm the lack of phenotype-expression correlation across diets. These lines were selected to represent the diversity of dietary reaction norms for triglyceride levels on control and high fat diets, and included seven lines from the original 20 assayed by microarray, with significant variation in triglyceride levels on the control and high-fat diets. Samples for TAG analysis and RNA profiling were collected from third instar larvae from three or more pooled food vials for each genetic line at two independent time point replicates raised in the same manner described previously and fasted for 1−2 hr. Two samples of 10 larvae were used for the lipid analysis, whereas the remaining larvae were used for expression analysis. RNA was extracted as described previously and then sent to Expression Analysis Inc. for quantitative reverse-transcription polymerase chain reaction (Q-RT-PCR) analysis using the Fluidigm BioMark platform. Forty-eight genes, including two housekeeping genes (Gapdh1 and Act5C), were assayed using custom Fluidigm DeltaGene assays (Table S12). One target gene failed to amplify in some samples, resulting in a total of 45 target genes with complete data, from 45 genetic lines on two diets replicated at two independent times, which totaled to 180 samples (File S8).

### Data analysis

All statistical analyses were performed using JMP Genomics. The standardization and normalization of the metabolomic and whole-genome expression data sets is presented in Reed *et al.* (2014).

To identify metabolite and gene expression profiles that differed significantly for the effects of diet, genetic line, and the genetic-by-diet interaction, we used the linear model:

$$Y_{ijm}=\mu +G_{i}+D_{j}+G\times D_{ij}+\varepsilon_{ijm}$$

for measurements taken from the *m*th individual sample in the *i*th genetic line (*G*) raised on the *j*th diet (*D*).

All results described as “significant” for individual genes and metabolites are at a false discovery rate of 0.05 or less unless otherwise noted.

Correlations among gene expression, metabolites, and gross phenotype were calculated from the least-squared means for each gene, metabolite, and gross phenotype grouped by genetic line, diet, and replicate. Forward stepwise regression was performed for each gross phenotype against the metabolites and transcripts shown to have significant pairwise correlations with a given phenotype, using default settings in JMP. A Bayesian information criterion was used for model selection.

#### Modulated modularity clustering analysis:

An analysis to determine coexpressed genes to identify possibly functionally relevant biological modules was performed with modulated modularity clustering (Stone and Ayroles 2009) on sets of genes filtered by statistical correlation with a biological factor of interest (*e.g.*, genes with significant genotype-by-diet interactions or those highly correlated with weight). Genes were filtered at a correlation significance level of *P* < 0.01 (Harbison *et al.* 2009) unless otherwise noted. The gene lists identified as occupying correlated expression modules via this method were subsequently queried against the Gene Ontology (GO) database with the use of the default settings in DAVID (Huang *et al.* 2009a,b) and filtered for an enrichment score of 1.3 or greater. The Kyoto Encyclopedia of Genes and Genomes (KEGG; http://www.genome.jp/kegg/pathway.html) pathway enrichment was also assessed with DAVID.

### Data availability

The dataset supporting the results of this article is available in the GEO database repository accession number GSE50745, http://www.ncbi.nlm.nih.gov/geo/query/acc.cgi?acc=GSE50745.

## Results

In previous studies, we observed highly significant genetic, dietary, and genotype-by-diet variation for each of the gross phenotypes of weight, total sugar, and triglycerides, as well as in whole-genome expression profiles and metabolomic profiles and found that the genotype-by-diet interaction effect explained a surprisingly large portion of the total phenotypic variance (Figure S1) (Reed *et al.* 2010, 2014). Building from the dataset originally reported in Reed *et al.* (2014), here we have presented the analyses that specifically focus on the complex genotype-by-diet interaction effects on MetS-like symptoms that can be modeled using this multidimensional dataset with in-depth exploration of the variance profiles of the individual phenotypes of gene expression and metabolite levels. We address how the genetic variation in the gross phenotypes of body weight, triglyceride storage, and total sugar (glucose and trehalose) are linked to metabolite profiles and gene expression profiles across four different diets. We also test how well gene expression correlations with phenotypes across distinct environmental conditions and populations can be replicated.

### How are genotype-by-diet interaction effects reflected in metabolomic and gene expression profiles, and do these profiles inform prediction of genotype-by-diet effects on MetS-Like phenotypes?

We analyzed individual gene expression levels for the contribution of genetic, dietary, and genotype-by-diet effects. The lists of individually significant genes can be found in File S1. Significant genetic effects were present for 9908 transcripts (85% of the expressed transcripts), and those genes showed significant (negative log_10_ p-value NLP > 4) GO enrichment for the functions of oxidation-reduction and peptidases (Table S1). Correspondingly, the KEGG pathways for the metabolism of xenobiotics by cytochrome P450s, degradation of limonene and pinene, drug metabolism, glutathione metabolism, and retinol metabolism all showed significant enrichment (*P* < 0.05, Table S1).

For diet effects, 311 transcripts were significant, and their GO enrichment clusters included phospholipase activity, choline kinase-like, and oxidation-reduction (Table S1). KEGG pathways enriched in the significant diet effect transcripts included glycerophosolipid metabolism, limonene and pinene degradation, alpha-linolenic acid metabolism, tyrosine metabolism, and biosynthesis of unsaturated fatty acids (Table S1).

**Figure 1:**
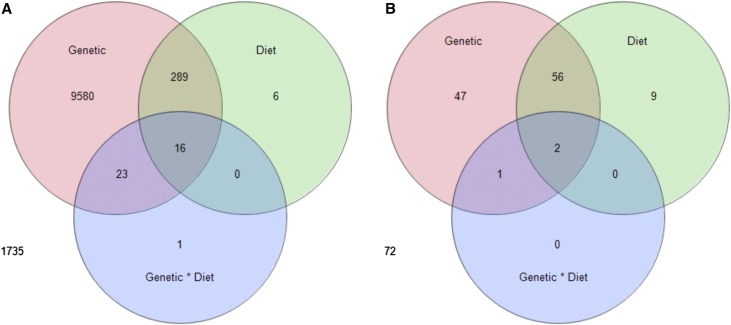
Transcripts and metabolites significant for genetic, dietary, and genotype-by-diet interaction effects. Number of significant transcripts (n = 219) of 11,650 (A) and metabolites (n = 425) out of 187 (B) determined by at a threshold of false-discovery rate = 0.05. Most genotype-by-diet significant transcripts and genes also were significant for main genetic and/or dietary effects. Numbers outside of the Venn diagrams represent the number of metabolites or transcripts that were not significant for any of these effects.

The genotype-by-diet effect was significant for 40 transcripts. The reduced proportion of significant transcripts relative to the overall large genotype-by-diet effect in the variance partition was largely due to the low sample size, thus reduced power for each genotype-by-diet combination. No KEGG pathways showed significant enrichment for genotype-by-diet effects. The number of genes sharing significant genetic, dietary, and genotype-by-diet effects can be seen in Figure 1A.

For individual metabolites, 106 showed significant genetic effects (56.6% of the measured metabolites), and 67 showed significant dietary effects (Figure 1B, File S2). The identified metabolites with the greatest genetic variation were leucine, isoleucine, and maltose (NLP > 20; File S2, Figure S2). However, the majority of metabolites tested in the categories of amino acids, amines, saturated fatty acids, steroids, and disaccharides showed significant genetic effects (Table S2). The identified metabolites with the strongest effect of diet were the saturated fatty acids with 12-carbons (12c), 14-carbons (14c), and 16-carbons (16c) (NLP >23; File S2, Figure S2). Seven metabolites were significant for the genotype-by-diet interaction effect (*P* < 0.01), including four fatty acids (12-, 14-, and 16-carbon saturated and 16-carbon unsaturated-palmitoletic), two branch chain amino acids (leucine and isoleucine), and adenosine (a building block of ATP, Figure 1B, Table 1, and Figure S2). The diversity of diet-induced reaction norms is exemplified in a group of 10 genetic lines that exhibited an increase in dodecanoic (lauric) acid specifically on the high-sugar diet (Figure S2), demonstrating a diet-specific genetic effect present at a high frequency in the population of sampled genetic lines.

**Table 1:** Metabolites with significant genotype-by-diet interaction, *P* < 0.01

Target	Likely Category	Confirmed ID
Target_0149	Amino acid	Leucine
Target_0164	Amino acid	Isoleucine
Target_0335	Saturated fatty acid	Dodecanoic acid
Target_0428	Saturated fatty acid	Tetradecanoic acid (myristic acid)
Target_0511	Unsaturated fatty acid	Palmitoletic acid
Target_0521	Saturated fatty acid	Hexadecanoic acid (palmitic acid)
Target_0779	Nucleoside	Adenosine

**Figure 2:**
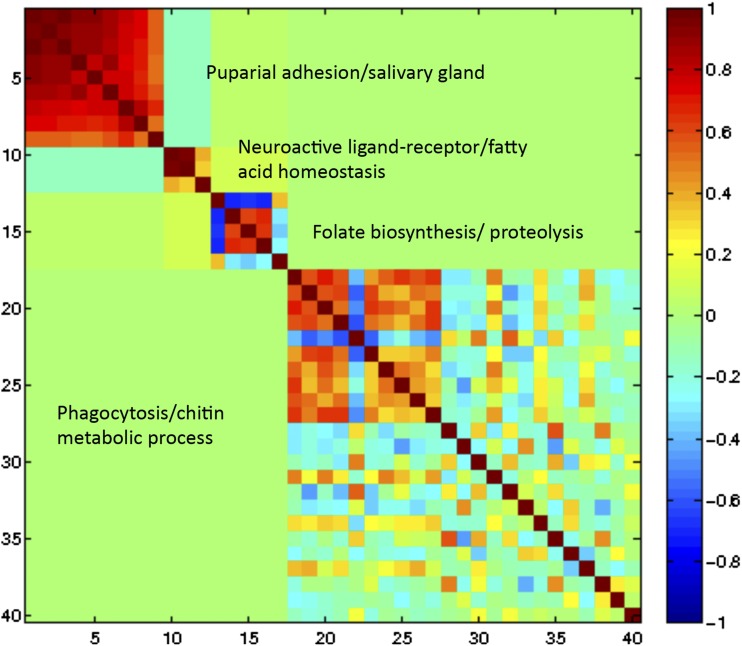
Modularity clustering of 40 transcripts significant for a genotype-by-diet interaction effect produces four distinct modules. Modules one through 4 are ordered from left to right. A total of 11,650 transcripts were tested and significance was determined at false-discovery rate = 0.05 (n = 219). Transcripts are grouped by correlated expression pattern, with red indicating a positive correlation and blue a negative correlation. Gene Ontology term enrichment is provided next to each module. The most strongly intercorrelated module (module one) was enriched for puparial adhesion and salivary gland gene function.

We clustered the 40 transcripts found to be significant for the genotype-by-diet effect using the Modulated Modularity Cluster (MMC) algorithm on correlations (Stone and Ayroles 2009), resulting in four modules of highly correlated transcripts (Figure 2 and Table S3). Of the nine genes occupying module one, five had associated GO function in puparial adhesion (Table S3). For module one, the first principal component (PC1) was correlated with the gross phenotype of pupal weight (*P* < 0.0001) and total sugar (*P* = 0.0112). There was a trend for significance in PC1 with triglycerides (*P* = 0.051). The tight correlation in expression patterns and gross phenotypes provides evidence that this particular suite of genes may be especially useful in explaining the mechanistic link between these MetS-like phenotypes.

**Figure 3:**
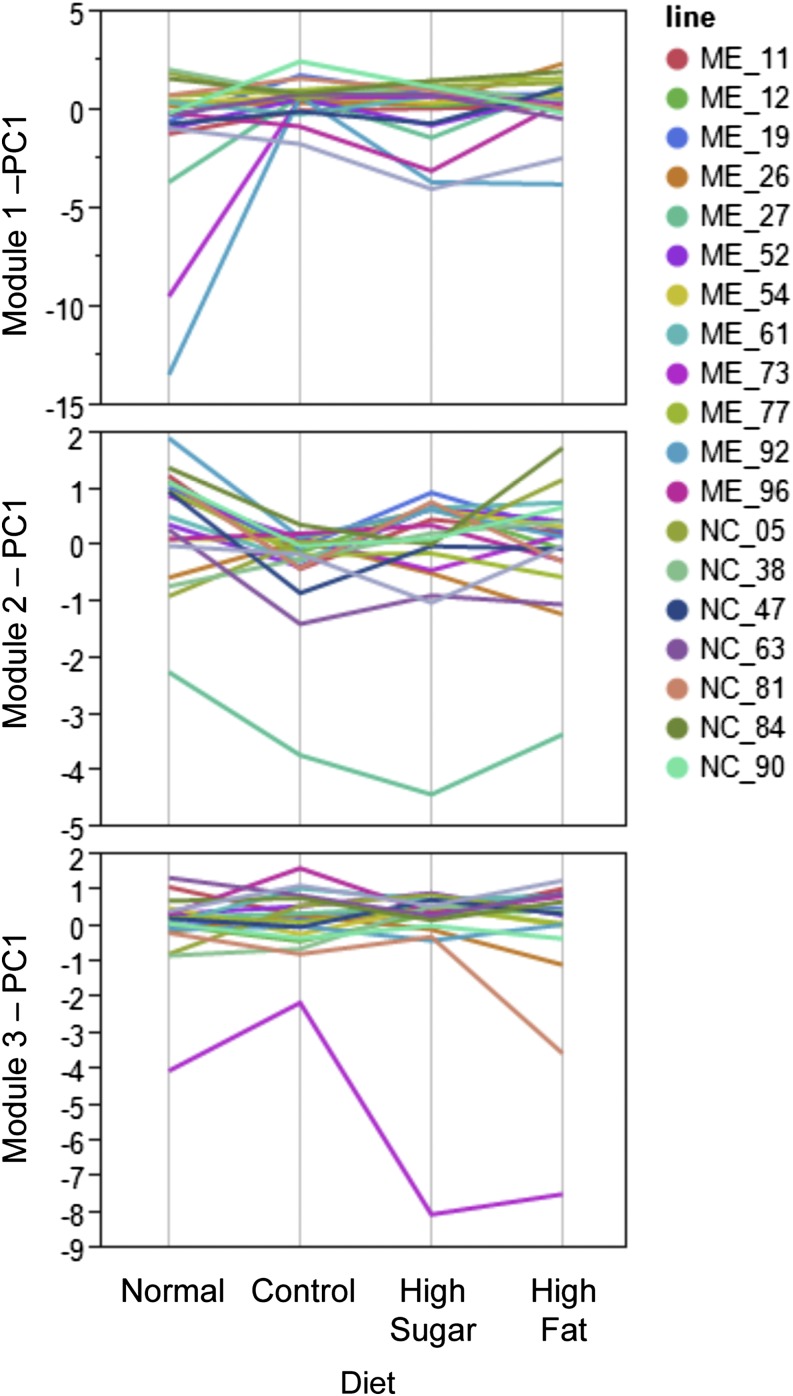
Genotype-by-diet interaction effects for significant transcript modules. Modules are those shown in Figure 2 and Table S1. Genotype-by-diet−specific loadings for the first principle component (PC1) of each module are graphed here. Each colored line corresponds to an inbred isofemale line. Notice that the PC1 for module 2 separates a single phenotypically distinct line, module 3 indicates two distinct lines, whereas the PC1 of module one can distinguish three genetic lines in a diet-specific manner.

The remaining three modules in the MMC analysis of the significant genotype-by-diet transcripts did not show an overall correlation across diet with any of the gross MetS-like phenotypes, although some did show diet-specific correlations. The second module was correlated with triglyceride variation on the high-sugar diet (*P* = 0.0359) (Figure S1). Module three was correlated with triglyceride levels on the control (*P* = 0.0137) and high-sugar (*P* = 0.0181) diets, which are the two diets that produced a notable reduction in larval triglycerides levels (Figure S1). In general, genes that showed significant genotype-by-diet interaction effects have the potential to help explain both the common mechanisms for genotype-by-diet effects across the MetS-like phenotypes (*e.g.*, module one), as well as the ways the phenotypes may also have independent mechanistic links to genetic and dietary variation (*e.g.*, modules two and three). The genetic line-specific reaction norms for PC1 of each of the modules can be seen in Figure 3. The PC1 of module one showed three lines to be distinct from the remaining 16 genetic lines with reduced values on the normal and high-sugar diets. For modules two and three, different individual genetic lines demonstrated themselves to be distinct from the rest of the population for their PC1 reaction norms. Line-specific reaction norms for expression principal components indicate that genetic mapping of causative loci for diet-specific expression quantitative trait loci could be feasible.

### Which endophenotypes link the gross metabolic phenotypes?

#### Gene expression by gross phenotype:

Pupal weight was significantly correlated with 479 transcripts enriched for the GO categories of puparial adhesion, regulation of protein import into the nucleus, and iron ion binding (Table S4 and File S3). The KEGG pathways showing enrichment in the transcripts correlated with weight include Insect Hormone Biosynthesis and One Carbon Pool by Folate (Table S4 and File S3). Triglycerides were correlated with 35 genes and had GO functional category enrichment for instar larval or pupal morphogenesis (Table S4 and File S3). In addition, the two linked KEGG pathways of inositol phosphate metabolism and phosphatidylinositol signaling system both showed enrichment in the genes correlated with triglycerides due to the shared genes *skittles* and *synaptojanin*. Total sugar was correlated with 82 transcripts that were significantly enriched for the GO function of oxidation-reduction (Table S4 and File S3).

Using forward stepwise regression, we asked how much of the variation in the gross phenotypes could we explain with independent transcripts (Table S5). There were eight genes that explained 56% of the variation in weight, five genes explaining 33% of triglyceride variation, and four genes explaining 27% of sugar variation. This means a relatively small number of gene transcripts had the ability to explain a large portion of the variance in metabolic phenotypes across variable genetic populations and diets. When examining the functional annotations of this subset of genes, we found there was no enrichment for any particular function, which is consistent with each gene reflecting a distinct component of the organism’s physiological influences on these traits.

#### Metabolites by gross phenotype:

For individual metabolites, 30 had a significant correlation with weight (Table S6), dominated by amino acids (9) and also included fatty acids (3) and sugars (3). Triglycerides were correlated with 12 metabolites, including amino acids (3) and sugars and sugar alcohols (4), but surprisingly not fatty acids, whereas total sugar was correlated with 14 metabolites, including amino acids (3), carboxylic acids (2), and sugars and sugar alcohols (3), but once again no fatty acids (Table S6).

We were then interested in whether a subset of metabolites could explain a significant portion of the variation in the phenotypes using forward stepwise regression. We found that two metabolites explained 18.6% of the variance in weight, five metabolites explained 17.4% of the variance in triglycerides, and two metabolites explained 10.2% of the variance in total sugar (Table 2 and Table S7). This subset of nonredundant metabolites represents distinct chemical groups and biological functions. For example, the five informative metabolites for triglycerides consist of two neurotransmitters, an amino acid, a sugar alcohol, and a polysaccharide.

**Table 2:** Metabolites significant in a stepwise regression with gross phenotype

	Weight		Triglyceride		Sugar	
	Sugar alcohol	Target_0678	*Arachidonoyl dopamine*	Target_0791	Cadaverine	Target_0426
	Hexadecanoic acid	Target_0472	Scyllo-inositol	Target_0524	Nonamide	Target_0595
			Catecholamine-like	Target_0526		
			*Glycine*	Target_0074		
			Pentasaccaride	Target_1029		
R-squared		0.186		0.174		0.102

Numbers in italics indicate confirmed ID.

#### Metabolite and gene expression correlations across gross phenotypes:

**Figure 4:**
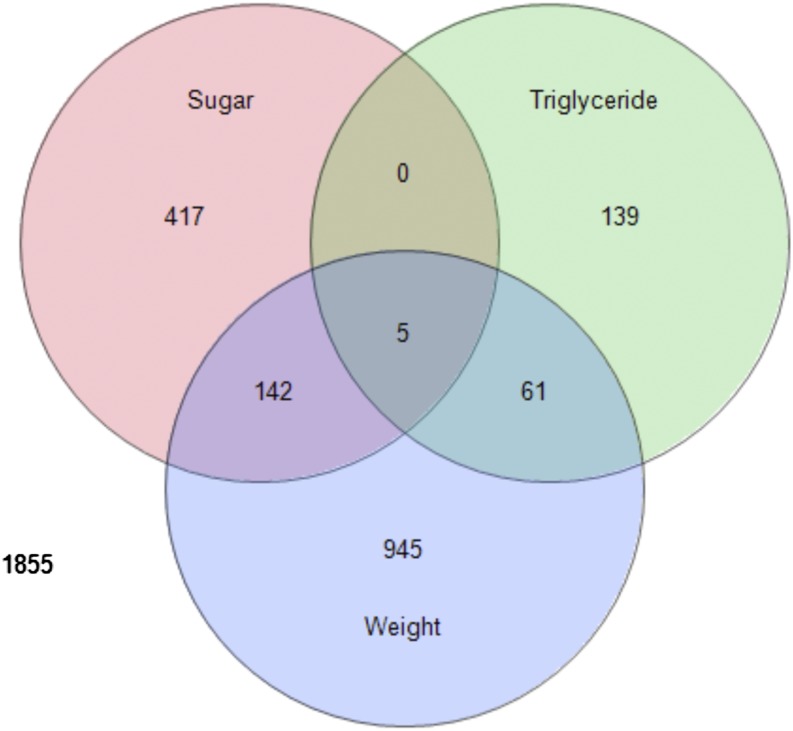
Transcripts significantly correlated with weight, triglycerides, or sugar. Significance determined at *P* < 0.01 (n = 80). Only five transcripts of 11,650 possible transcripts were correlated with all three traits, which is surprising given the correlations among these gross phenotypes, and suggests the intertrait correlations may not be driven by gene expression variation.

We were interested in identifying endophenotypes that potentially could help to create new mechanistic links between the different MetS-like phenotypes in the flies. We initially asked whether any of the genes correlated with multiple phenotypes. We found 147 genes in common between the weight and sugar phenotypes (*P* < 0.01), 66 genes in common between weight and triglycerides (*P* < 0.01), and 93 between triglycerides and sugar (*P* < 0.05, Figure 4 and Table S8). One intriguing set of three genes in common to triglyceride and sugar phenotypes was purported to be involved in neuropeptide signaling, which may indicate a behavioral or hormonal link between these traits. Of the five genes that were common to all three phenotypes (Figure 4 and Table 3), two had no previously described function, and the remaining three had no obvious link to metabolic function, but one of the three genes was associated with histone acetylation. The lack of profound functional enrichment of the common set of genes suggests that there is much unexplored territory for new mechanistic links between MetS traits.

**Table 3:** Genes significant for weight, triglyceride, and sugar (*P* < 0.01)

Transcript ID	Gene ID	Molecular Function	Biological Process
FBtr0073750	CG12715	Unknown	Unknown
FBtr0076215	CG14143	Unknown	Unknown
FBtr0077911	fritz	Unknown	Imaginal disc-derived wing hair site selection; establishment of planar polarity
FBtr0084628	CG18528	GTP binding; GTPase activity	tRNA modification; GTP catabolic process
FBtr0100273	Rpb4	Protein binding; DNA-directed RNA polymerase activity; chromatin binding	Histone H3 acetylation; cell proliferation; mitotic cell cycle G2/M transition DNA damage checkpoint; histone H4 acetylation; transcription from RNA polymerase II promoter; neurogenesis

When testing for correlations between the gross phenotypes, we found that raw values were positively correlated between weight and triglycerides (NLP = 2.11; Table 4), but the correlations among the other phenotype pairs were not significant. Given the general pattern of correlation among these traits in other systems, we were led to ask whether there was a signature of correlation among these phenotypes within metabolite profiles or expression profiles. We found that the correlation coefficients between metabolites and weight were positively correlated with the correlation coefficients between metabolites and triglycerides (NLP = 6.28; Table 4), meaning that metabolites expressed at high levels in heavy flies also were expressed at high levels in flies with high triglyceride levels, and vice versa. In fact, we found that for all three traits there was a highly significant co-correlation between metabolites and transcripts for each trait. Co-correlations of metabolites and transcripts with weight and sugar, and between triglycerides and sugar, were highly negative, whereas metabolites and transcripts were positively co-correlated between weight and triglycerides (Table 4). This means that even when there was no significant correlation among the gross phenotypes (*e.g.*, R = −0.092 for weight and total sugar), there could be an enormous co-correlation among the endophenotypes (*e.g.*, R = −0.697 for transcript co-correlation between weight and sugar, Table 4).

**Table 4:** Phenotype correlations by raw, metabolite, and gene expression correlates

Phenotype 1	Phenotype 2	Raw Phenotypes			Phenotypes by Metabolite Correlation			Phenotypes by Expression Correlation		
		Correlation	Count	NLP	Correlation	Count	NLP	Correlation	Count	NLP
Triglyceride	Weight	0.181	217	2.11	0.357	187	6.28	0.341	11650	312.0
Sugar	Weight	−0.092	212	0.74	−0.550	187	15.43	−0.697	11650	312.0
Triglyceride	Sugar	0.059	205	0.40	−0.456	187	10.27	−0.429	11650	312.0

NLP, negative log_10_ p-value.

**Figure 5:**
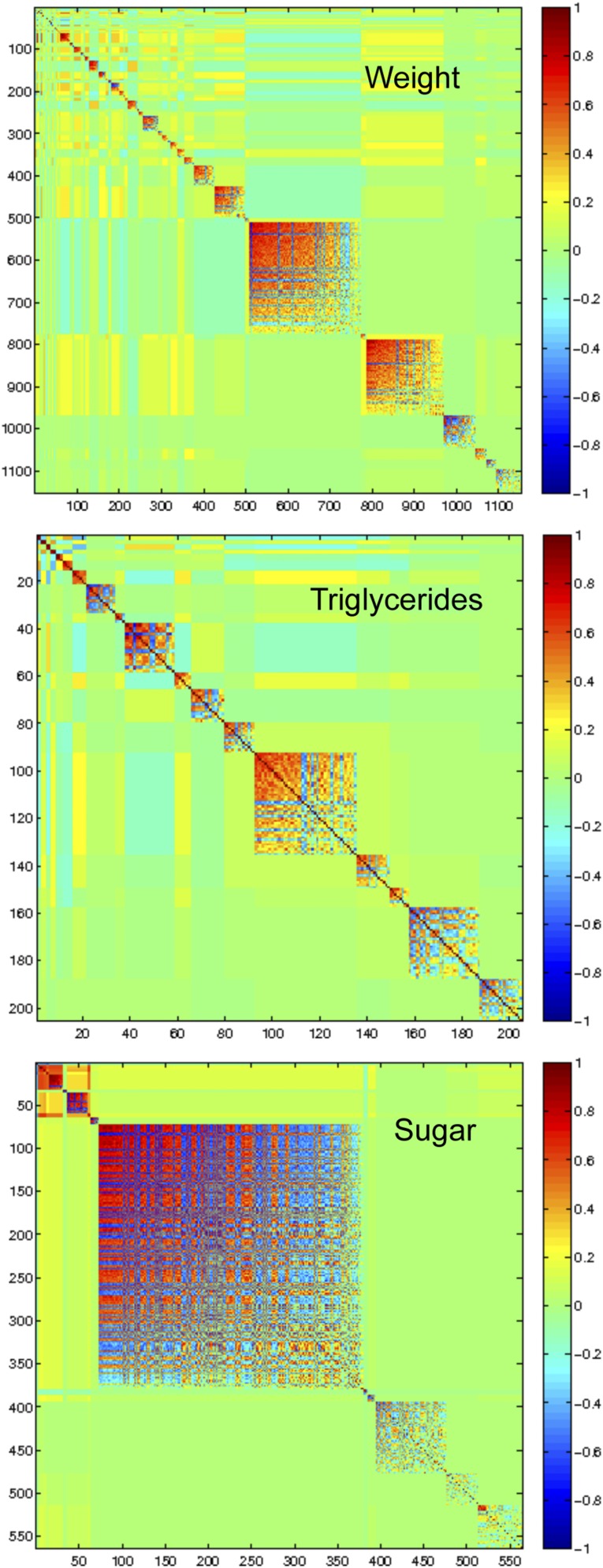
Modularity of transcripts significantly correlated with weight, triglycerides, and total sugar. Strong modularity of expression-phenotype correlates is apparent. Significance determined at *P* < 0.01 (n = 80). Transcripts are grouped by correlated expression pattern, with red indicating a positive correlation and blue a negative correlation. Modules are numbered from left to right, and the composition is given in Table 5 and File S4.

Given the strength of the co-correlation of endophenotypes, we next asked whether we could parse these broad correlations in a way that could help identify the underlying mechanisms. To address this, we performed MMC analysis on the genes significantly correlated with each gross phenotype (weight, triglycerides, and sugar) at a significance threshold of *P* = 0.01 (1154, 206, and 565 transcripts respectively, File S3) and identified modules of genes with strong correlation in expression pattern (Figure 5). For weight, we found six modules, containing 15 or more transcripts with an average degree of correlation >0.5, four of which also correlated with sugar (Table 5). The genes in weight module 25 were significantly correlated with sugar and were enriched (NLP > 12; Table 5) for the GO function category puparial adhesion. For triglycerides, there were three modules, with three or more transcripts, and module five also correlated with weight and sugar (Figure 5, Table 5, File S4). Because triglyceride module five correlates with all three gross phenotypes, its correlated genes, which include a probable cytochrome P450, *Cyp313a4*, and a signal transduction gene, *CG32082*, are particularly exciting and in need of further functional experimentation and validation.

**Table 5:** Modulated modularity clustering analysis of genes correlated with traits

Trait Module	Number of Transcripts	Average Degree of Correlation	Significance of Module PC 1 Correlation With Trait			Top GO Category/*Named Gene(S)*	KEGG
			Weight	Triglyceride	Sugar		
Weight 25	23	0.612	****		*	Puparial adhesion (6, 1.9E-12)	
Weight 28	15	0.580	***		*	Regulation of protein polymerization (2, 2.2E-2)	
Weight 32	25	0.544	****		*	Macromolecular complex assembly (3, *ns*)	
Weight 33	16	0.535	****		**	Cytochrome P450 (2, *ns*)	
Weight 36	20	0.521	****			Proteasome regulatory particle, lid subcomplex (2, 7.3E-3)	Proteosome (2, *ns*)
Weight 40	22	0.501	***			Glycerol-3-phosphate metabolic process (2, 9.0E-3)	
Triglyceride 5	3	0.713	***	***	**	*Cyp313a4*	
Triglyceride 6	4	0.608		*		Posttranslational modification, protein turnover, chaperones (2, *ns*)	
Triglyceride 7	6	0.564		**		RNA processing (4, 5.7E-4)	
Sugar 6	17	0.758			***	Oxidation reduction (7, 5.3E-5)	Oxidative phosphorylation NADH dehydrogenase Complex 1 (4, 4.3E-3)
Sugar 7	4	0.725			***	*GSTE6/bloated tubules*	
Sugar 8	25	0.685	**		***	Generation of precursor metabolites and energy (5, 9.8E-4)	Citrate cycle /TCA cycle(4, 7.0E-4)
Sugar 9	4	0.652	**		***	*Collagen type IV*	
Sugar 10	9	0.612			***	Oxidation reduction (2, *ns*)	

GO, Gene Ontology; KEGG, Kyoto Encyclopedia of Genes and Genomes; TCA, tricarboxylic acid, * p<0.05, ** p<0.01, *** p<0.001, **** p<0.0001.

For sugar, there were five correlated modules with four or more genes. Sugar module six was highly enriched for oxidation-reduction function, containing four genes that code for components of Complex 1 of the electron transport chain. Sugar module eight, which was also significantly correlated with weight, was highly enriched for enzymes in the tricarboxylic acid cycle (Figure 5, Table 5, File S4).

### Which endophenotypes are predictive of gross phenotype in different dietary conditions?

#### Endophenotype correlations with gross phenotypes across diets:

**Figure 6:**
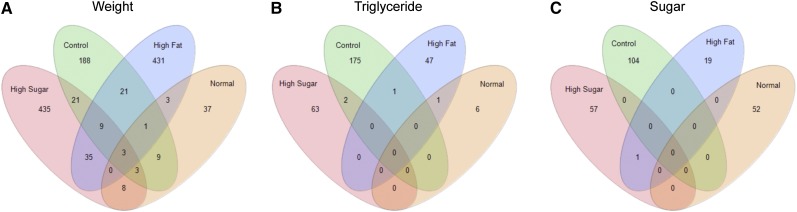
Transcripts significantly correlated by diet with weight, triglycerides, or sugar. Significance determined at *P* < 0.01 (n = 80). A surprisingly low number of transcripts of 11,650 were found to be significant for more than one diet for weight (A), triglycerides (B), and total sugar (C).

The final question we wanted to address was whether gene transcripts or metabolites that were correlated with a particular gross phenotype in one environment (diet) would remain correlated in other environments. Collectively, the overwhelming answer was no, there was no greater sharing of phenotype-correlated genes across diets than expected by chance alone. Of the 1180 transcripts that were correlated with weight on one or more diets, 1091 of them were unique to a specific diet, and only three were significantly correlated with weight across all four diets, whereas the pattern was even starker for triglyceride- and sugar-diet-specific correlates (Figure 6).

Despite the lack of conservation of significant specific transcripts correlated with phenotype across diet, we asked whether there was any evidence of a conservation of gene function. We observed some slight evidence of shared function in a few specific cases largely driven by the few genes that were in common to more than one diet (Table S9 and File S5), but observed no generally compelling pattern. One category of gene transcripts that might be useful in predicting weight across diets was related to nucleosome organization and regulation of histone modification (Table S9, Table S10, and Table S11 and File S5).

**Figure 7:**
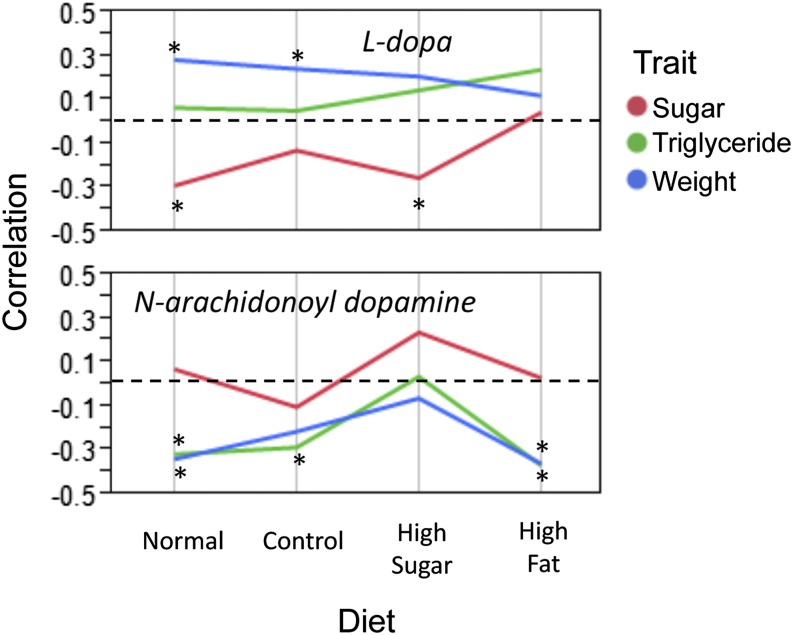
Selected metabolites showing strong correlation with weight, triglycerides, or total sugar. Values above the dashed line at 0 indicate a positive correlation, while values below the dashed line indicate a negative correlation. *Indicates a significant correlation at *P* < 0.01 (n = 80). L-DOPA was positively correlated with weight and negatively correlated with sugar and uncorrelated with triglycerides. *N*-arachidonoyl dopamine was negatively correlated with both weight and triglycerides on all but the high fat diet, but not significantly correlated with sugar.

The same pattern holds for metabolites. Using a significance cutoff of *P* < 0.01 for metabolites correlated with a phenotype on a diet, we found that only four of the 23 metabolites correlated with weight were correlated on more than one diet (File S6). Of the nine correlated with triglycerides, one was correlated on more than one diet, and for total sugar, none of the 11 metabolites were correlated on more than one diet (File S6). There were, however, a few metabolites that stood out as being plausible candidates for explaining phenotypic variance across diets. More than one phenotype showed diet-specific correlation between phenotype and metabolite for nine metabolites (*P* < 0.05, Figure S3). L-DOPA, a metabolic precursor to neurotransmitters such as dopamine, was correlated with weight on the normal and control diets, and correlated with total sugar on the normal and high-sugar diets, but was uncorrelated with triglycerides (Figure 7). Thus, L-DOPA provides a good candidate for a neurological link between weight and total sugar, as modulated by diet. Furthermore, *N*-arachidonoyl dopamine provides a very enticing mechanistic link between dietary influenced triglycerides and weight because it was correlated with triglycerides and weight on the normal and high fat diets, as well as with triglycerides on the control diet (Figure 7). These two metabolites also were detected in our original analyses (Reed *et al.* 2014) as having strong loadings in the second principal component of the overall metabolite profiles, which was the principal component that correlated with multiple gross phenotypes (weight, triglycerides, sugar, and heart arrhythmia index).

#### Population-specific effects:

After observing the startling lack of conservation of transcripts correlated with phenotype on different diets, we asked whether this was a general pattern or a phenomenon specific to the subset of genetic lines used in the initial study. We performed a follow-up experiment where 45 genetic lines, which were selected from among those studied in Reed *et al.* (2010) to represent the diversity of reaction norms for triglyceride response to diet, were raised on normal and high fat diets. In the original dataset, the correlation of the correlation coefficients between the high fat and control diets was a negligible 0.0188 (*P* = 0.90, Table 6). However, the correlation of transcript-by-trait correlation coefficients between the two diets in follow-up experiments was 0.8606 (*P* < 0.0001, Table 6). The differences in correlation and methods for measuring gene expression lead us to conclude that, as was already well-documented (Ayroles and Gibson 2006; Morey *et al.* 2006; Git *et al.* 2010), Q-RT-PCR analyses of gene expression are less susceptible to experimental stochasticity and have a greater dynamic range than microarray technologies, thus allowing for a more robust documentation of biological signal.

**Table 6:** Gene expression and triglyceride storage correlations across experimental methods and diet

Methods		Mircroarray		Quantitative PCR	
	Diet	Control	Fat	Control	Fat
Microarray	Control		0.0188 (-0.0588)	0.4867 (0.3379)	0.4195 (0.2859)
	Fat	0.9003 (0.6946)		−0.2501 (-0.4168)	−0.2914 (-0.5079)
Quantitative PCR	Control	**0.0005 (0.0202)**	0.09 (**0.0036**)		0.8606 (0.7299)
	Fat	**0.0033** (0.0514)	**0.0469 (0.0003)**	**<0.0001 (5.80E-09)**	

Correlations are shown above the diagonal and *P*-values below; values from subset of common genetic lines are in parentheses, values significant at p<0.05 indicated in bold. PCR, polymerase chain reaction.

We then asked whether the correlations between triglyceride levels and these transcript-by-diet correlation coefficients in the main microarray experiment was predictive of the correlations found in the Q-RT-PCR follow-up. We found that the co-correlation between the mircroarray control diet and the Q-RT-PCR control and high-fat diet was both positive and substantial (Table 6). However, although triglyceride-transcript correlations on a control diet were predictive of triglyceride-transcript correlations in a Q-RT-PCR experiment, the results from the high-fat diet were not (Table 6).

Puzzled by these findings, we next asked whether this pattern was true for the subset of seven genetic lines that were used in both experiments, and we found that all of the general patterns described previously remained the same (Table 6). These results led us to conclude that the patterns of correlation in gene expression with phenotypes in a subset of a genetic population can be predictive of trends in the larger population.

## Discussion

It is clear from this study that both metabolite profiles and expression profiles are strongly influenced by genetic and genotype-by-environment interaction effects and that diet-specific effects are particularly well detected through metabolite profiles. Because gene expression patterns and metabolomic profiles also react in different ways to variation in diet and genotype, they each provide distinct perspectives regarding the mechanisms linking environment and genotype to gross phenotypes.

### Patterns in genotype-by-diet Interactions

Analyses of the subset of transcripts and metabolites that showed a significant genotype-by-diet interaction effect are especially profitable for identifying potential mechanisms influencing the genotype-specific reactions to dietary changes observed for symptoms of metabolic syndrome.

#### Transcript genotype-by-diet interaction:

The first module from the MMC analysis of the transcripts significant for a genotype-by-diet interaction effect was a powerful example of how parsing the correlational data into modules exposes underlying function. Each of the gross phenotypes of weight, triglycerides, and total sugar was significantly or nearly significantly correlated with this module, and eight of the nine genes in the module were involved in puparial adhesion function, suggesting that this is something linking the pathways used to prepare a larvae for pupation with these metabolic symptoms.

We also found that expression of three genes in the insect hormone biosynthesis pathway, Juvenile hormone esterase (*Jhe*), phantom (*phm*), and shadow (*sad*), were positively correlated with pupal weight in our dataset, whereas *sad* also was negatively correlated with sugar. Specific pathways associated with metamorphosis have already been well characterized in flies in relation to body size and development, such as those mediated by ecdysone and juvenile hormones (Flatt and Kawecki 2007; De Loof 2008; Mirth *et al.* 2009; Tennessen and Thummel 2011; Riddiford 2012; Schwedes and Carney 2012; Koyama *et al.* 2013; Danielsen *et al.* 2013; Yamanaka *et al.* 2013), thus further confirming that these expression patterns are useful signals. From this study, we cannot tell which aspects of the ecdysone/puparial adhesion relationship with MetS are causal and which of the effects are correlated responses to an earlier developmental effect to genotype-by-diet interaction. However, knowing that genes associated with pupariation and metamorphosis in flies also are related to metabolic health in flies greatly focuses our future explorations for causal mechanisms.

#### Metabolite genotype-by-diet interaction:

Focusing on the metabolites with a significant genotype-by-diet interaction effect also enabled us to identify a subset of metabolites that may be especially important in mediating an organism’s physiological reaction to a change in environment. We found that the branched chain amino acids (BCAAs), leucine and isoleucine, had strong genotype-by-diet effects and were highly correlated with each other (*P* < 0.0001). These two metabolites also had strong loadings on the second principal component of the overall metabolite profile that correlates with weight, triglycerides, sugar, and arrhythmia index (Reed *et al.* 2014). BCAAs have been associated with heart disease risk, gestational diabetes, and obesity in metabolomic studies in humans (Zeanandin *et al.* 2011; Johnson *et al.* 2013; Patel *et al.* 2013; Bhattacharya *et al.* 2014; Scholtens *et al.* 2014).

Leucine is used in the synthesis of sterols in adipose and muscle tissue (Rosenthal *et al.* 1974; Combaret *et al.* 2005), and it has the ability to stimulate the synthesis of muscle protein (Aftring *et al.* 1986; Zeanandin *et al.* 2011). In addition, increased leucine doses to the rat brain have been shown to decrease body weight through reduced food intake by activating the TOR signaling pathway (Mensink *et al.* 2003; Combaret *et al.* 2005; Cota *et al.* 2006).

Isoleucine is gluconeogenic, as it can be broken down to succinyl CoA and then used by the tricarboxylic acid cycle, as well as functional in ketosis by being converted into acetyl CoA and then synthesized into fatty acids (Aftring *et al.* 1986; Doi *et al.* 2007). Isoleucine also was correlated significantly with weight in our dataset. Because BCAAs play a hub-like role in energy homeostasis, they are excellent targets for potential MetS treatments as well as a proof-of-principle that a systems biology approach of MetS in flies does recover pathways relevant to mammalian systems.

The four fatty acids showing a significant genotype-by-diet interaction were between 12c and 16c in length. These interaction effects remained significant for the 12c and 14c fatty acids, even when the data from the high-fat diet were excluded from the analysis, suggesting that increased exposure to fatty acids in the diet was not sufficient to explain these correlations.

Dietary lauric acid (12c) is associated with an increase the “good” HDL cholesterol in mammals and thus likely decreases atherosclerotic risk (Mensink *et al.* 2003; Fattore and Fanelli 2013). Palmitic acid (16c), when fed to rats, has been shown to suppress their leptin and insulin signaling systems (Benoit *et al.* 2009*)*, leading to increased appetite. In addition, excess consumption of palmitic acid is thought to increase the risk of cardiovascular disease (Cao *et al.* 2008; Fattore and Fanelli 2013).

In contrast, palmitoletic acid, a monounsaturated 16c fatty acid, increases insulin sensitivity (Yang *et al.* 2011) and may influence fatty liver deposition, and has thus been dubbed a “lipokine” for having hormone-like effects (Cao *et al.* 2008). Palmitic acid also was correlated significantly with weight in our dataset, whereas myristic acid (14c), palmitic acid, and palmioletic acid were highly intercorrelated. The strong correlation between these metabolites and the MetS-like symptoms suggests that all of these metabolites are excellent candidates for mediating the genotype-by-diet effects of metabolic phenotypes.

### Patterns in endophenotype and gross phenotype correlations

Analyses focusing specifically on which transcripts and metabolites were correlated with multiple gross MetS-like phenotypes were also quite illustrative.

#### Transcript-gross phenotype correlation:

Gene transcripts correlated with both weight and triglycerides were enriched for oxidation-reduction function (which is involved in central energy metabolism, a critical component of maintaining energy homeostasis) and for the valine, leucine, and isoleucine degradation pathway (BCAAs involved in gluconeogenesis, steroid synthesis, and appetite control as discussed previously). In the analysis of coexpressed modules correlated with each of the gross phenotypes studied, we recovered some modules highly enriched for known important metabolic systems, such as for components of Complex 1 of the electron transport chain or for enzymes in the TCA cycle. Thus, as previously demonstrated, functionallylinked metabolic genes can be recovered on the basis of correlated expression pattern alone and then linked to specific metabolic traits. This provides proof of principle that modules lacking obvious previous functional information may contain very important functionally linked metabolic and disease genes.

We found many tightly correlated expression modules correlated with MetS-like phenotypes had little or no previous functional characterization. An example of one such model is triglyceride module five from the MMC analysis that is also strongly correlated with weight and sugar and contains only three genes, of which only one is annotated to have any putative function (cytochrome P450). These new modules will be especially intriguing for future follow-up due to their potential to help elucidate the new fundamental mechanisms linking phenotypes to genetic and environmental conditions.

#### Metabolite-gross phenotype correlation:

Two of the metabolites that were correlated with body weight and one other phenotype, L-DOPA and *N*-arachidonoyl dopamine (also with strong loadings on metPC2 correlated with weight, triglycerides, sugar, and arrhythmia index reported in Reed *et al.* 2014), are important neurological signaling molecules. Additionally, a third metabolite that correlated with both weight and triglycerides was a yet to be determined catecholamine-like compound. L-DOPA is the metabolic precursor to the neurotransmitter dopamine, and dopamine can be converted into N-arachidonoyl dopamine by fatty acid amide hydrolase. *N*-arachidonoyl dopamine, also known as NADA, is a recently discovered endocannabinoid (bisogno *et al*. 2000; Bellocchio *et al.* 2008). In mammals, NADA binds the neuronal cannabinoid receptor CB1 and is an agonist for the vanilloid receptor (TRPV1) (Ralevic 2003; Bisogno *et al.* 2005).

The endocannabinoid signaling system has been described as playing a very important role in appetite regulation and obesity in mammals (Bellocchio *et al.* 2008), but it has not been implicated previously in appetite control in invertebrates. However, because we saw in our studies that an endocannabinoid was correlated with weight and triglyceride levels in *Drosophila*, as were two other catecholamine-related metabolites, *Drosophila* may be a powerful tool to explore the link between appetite and metabolic disease as mediated through the endocannabinoid/catecholamine signaling system.

We also observed that gene transcripts correlated with NADA were significantly enriched for enzymes in the tyrosine synthesis pathway. The tyrosine pathway is responsible for the synthesis of L-DOPA and dopamine, which are precursors to NADA, thus demonstrating a logical confirmation of biological function recovered from correlational data. In addition, the genes highly correlated with L-DOPA include six that code for components of the electron transport chain, the mitochondrial system that drives ATP synthesis, thus making an obvious potential link between the metabolite and its impact on energy homeostasis in the organism.

### Endophenotype-gross phenotype links across diets

Finally, in attempting to ascertain which endophenotypes contribute to MetS-like phenotypes across multiple dietary conditions, we were surprised to find a significant lack of common actors. The genes and metabolites highly correlated with a phenotype on one diet were almost never the endophenotypes that were most correlated with the phenotype on a different diet. This places even greater importance on the few endophenotypes that do correlate with MetS-like symptoms on multiple diets. One such gene group, which correlated with weight on multiple diets, was enriched for nucleosome organization and thus may be involved in the epigenetic regulation of weight. Another example was *N*-arachidonoyl dopamine, which correlated with both weight and triglycerides on multiple diets.

There are important evolutionary implications for the general lack of conservation of correlated endophenotypes across diets and the inability of correlation patterns in one genetic sample to generalize to a second genetic sample on a perturbing diet. The canalization of the physiology of metabolism is apparent across genetically variable populations tested in an environment to which they are well adapted. However, the breakdown of physiological homeostasis in a novel environment exposes cryptic variation for disease thus helping to explain how maladaptive traits such as those of MetS can occur at a high frequency in otherwise well-adapted populations (Gibson and Reed 2008; Gibson 2009; Reed *et al.* 2010, 2014). This suggests that great caution needs to be exercised when drawing general conclusions about what endophenotypes in one experiment, treatment, or population can tell us about the physiological underpinning of phenotypes in another condition.

Returning to the original questions motivating this study, first, we found that metabolomic and gene expression profiles both inform prediction of genotype-by-diet effects on MetS-like phenotypes but in a modular fashion. Individual pathways implicated by these correlated endophenotypes frequently have genotype-specific, diet-specific, and phenotype-specific effects, allowing for the largely independent influences from disparate components of metabolic physiology. Forward stepwise regression identified a minimum set of genes or metabolites, each representing independent upstream physiologies, needed to explain the maximum amount of variation in the gross phenotypes. We were able to explain 10–56% of the variation in phenotype across the population with eight or fewer variables, providing a handful of endophenotypes that might be good for screening for metabolic disease, many of whom have no previous recognized association with metabolic disorders.

We also found that the correlation coefficients between endophenotypes and the gross phenotypes were more strongly correlated with each other across the population than the gross phenotypes were on their own. Thus, analyzing co-occurring traits through their co-correlated endophenotypes such as in genetic mapping experiments may be much more informative than analyzing the raw traits alone for identifying mechanistic genetic links. The increased power of metabolites to facilitate genetic mapping has already been demonstrated in human metabolite GWAS studies where genetic effects explaining 15−60% of the variation in metabolites levels can be detected (Adamski and Suhre 2013; Shin *et al.* 2014).

Second, some of the endophenotypes linking the gross phenotypes of body weight, triglyceride storage, and sugars include metabolites like L-DOPA and NADA, fatty acids, and BCAAs, as well as correlated gene expression modules associated with pupariation, ATP synthesis, and the TCA cycle. In addition, a number of genes and metabolites with no previous association with MetS are implicated as candidates for metabolic homeostasis control through this study.

Third, the patterns of endophenotype-by-gross phenotype correlations found in a genetically variable population in a stable environment (*i.e.*, control diet) can predict how a different genetic population will perform in a perturbing environment (*i.e.*, high-fat diet), but the performance in a perturbing environment of one genetic population has no predictive power of performance of a different genetic population in that same perturbing environment. Therefore, a novel environment can expose latent genetic variation for physiological traits that differentiates populations that was not detectable in a stable environment. This has broad reaching implications from whether analysis of endophenotypes of predisease individuals can be informative of disease risk after a change in lifestyle to how to best perform research linking endophenotypes to gross phenotypes in heterogeneous populations.

Overall, this study shows that a multitude of physiological mechanisms influence a population exhibiting variation for disease traits, some that are specific to the genetic or environmental context of an individual and others that transcend genetic and environmental influences. Parsing larger endophenotype datasets into correlated modules has the potential to identify genotypes with unique physiological mechanisms for reacting to changes in the environment. Thus, there are multiple ways to achieve similar phenotypic outcomes, and recognition of this phenomenon could lead to the accelerated identification of the rare genetic variants contributing to common disease.

This study illustrates the power of systems biology conducted in model organisms to provide new insights into how the physiology of complex disease with both genetic and environmental factors may be parsed into more tractable experiments for mammalian and human systems. If we can freely explore the enormous parameter space of these physiologies and test preliminary hypotheses with relatively low risk in an invertebrate model, we can investigate entirely new ways of thinking about problems and perhaps find new promising avenues on which to focus our research efforts and financial resources in humans, avenues we might never have stumbled upon if we limited our focus to just humans.

## Supplementary Material

Supporting Information
