# Supplementary material for: Metabolomic and Gene Expression Profiles Exhibit Modular Genetic and Dietary Structure Linking Metabolic Syndrome Phenotypes in Drosophila
Source: G3 (Bethesda). 2015 Nov 3;5(12):2817–29. doi: 10.1534/g3.115.023564 (PMC4683653; doi:10.1534/g3.115.023564)
Supplement: Supporting Information [file supp_5_12_2817__index.html]

Metabolomic and Gene Expression Profiles Exhibit Modular Genetic and Dietary Structure Linking Metabolic Syndrome Phenotypes in Drosophila — Supporting Information 

# Metabolomic and Gene Expression Profiles Exhibit Modular Genetic and Dietary Structure Linking Metabolic Syndrome Phenotypes in *Drosophila*

## Supporting Information for Williams *et al.*, 2015

**Files in this Data Supplement:**

- Figure S1 - Genetic, dietary, and GxD interaction effects for weight, triglycerides, and sugar. (.pdf, 673 KB)
- Table S1 - GO analysis for significant ANOVA transcripts. (.pdf, 35 KB)
- Table S2 - Distribution of metabolite categories with significant genetic, dietary, and genotype\*diet effects. (.pdf, 37 KB)
- Table S3 - Genes with a significant GxD interaction effect (FDR 0.05) (.pdf, 41 KB)
- Table S4 - GO analysis of transcripts correlated with traits. (.pdf, 36 KB)
- Table S5 - Stepwise forward regression of transcripts correlated with gross phenotypes. (.pdf, 39 KB)
- Table 6 - Correlations between traits and metabolites. (.pdf, 48 KB)
- Table S7 - Stepwise forward regression of metabolites correlated with gross phenotypes. (.pdf, 37 KB)
- Table S8 - GO analysis of genes overlapping traits. (.pdf, 37 KB)
- Table S9 - GO category enrichment by diet for weight, triglyceride, and sugar. (.pdf, 38 KB)
- Table S10 - Gene IDs for transcripts correlated with traits across multiple diets. (.pdf, 37 KB)
- Table S11 - GO category enrichment for genes correlated with weight on multiple diets. (.pdf, 36 KB)
- Table S12 - Primers used in Fluidigm Q-PCR analysis. (.pdf, 43 KB)
- Figure S2 - Metabolites with significant genotype-by-diet interaction effect and confirmed identities. (.pdf, 1,728 KB)
- Figure S3 - Metabolites showing strong correlation with weight, triglycerides, or total sugar. (.pdf, 836 KB)
- File S1 - Genetic, dietary, and genotype-by-diet interaction effects for individual genes. Column headings: Gene ID = Flybase Gene ID, Transcript ID = Flybase transcript ID, RSquare = coefficient of determination for model including genetics, dietary, and genotype-by-diet interactions, F-value xxxx = F statistic for a given effect, NLP xxxx = negative log probability for a given effect, FDR xxxx = False Discovery Rate for given effect, Significant at FDR 0.05 xxxx = whether FDR < 0.05 for a given effect. (.xlsb, 1,369 KB)
- File S2 - Genetic, dietary, and genotype-by-diet effects for individual metabolites. Column headings: Target Code = study-specific unique ID, Likely Category = general chemical class of compound, Likely ID = best NIST match for compound, confirmed ID = chemical standard confirms ID, RSquare = coefficient of determination for model including genetics, dietary, and genotype-by-diet interactions, F-value xxxx = F statistic for a given effect, NLP xxxx = negative log probability for a given effect, FDR xxxx = False Discovery Rate for given effect, Significant at FDR 0.05 xxxx = whether FDR < 0.05 for a given effect. (.xlsx, 39 KB)
- File S3 - Pairwise transcript-by-phenotype correlation. Variable = Flybase transcript ID, With = phenotype, Pearson\_Correlation =correlation coefficient, Nobs = sample size, NegLog10\_p = negative lobe probability for a significant correlation, NegLog10\_p FDR Adjusted = False Discovery Rate, Gene ID = Flybase gene ID. (.txt, 2,221 KB)
- File S4 - Modulated Modularity Clustering of genes correlated with gross phenotype. Worksheet Tabs: pupal weight (weight-siggenes), triglyceride (TG-siggenes), and trehalose (sugar\_siggenes). Columns: Gene = Flybase Gene ID, Module = module group from MMC analysis, Entry Index = arbitrary order of gene ID input, Average Degree = average pairwise correlation within module, Degree = average of pairwise correlation between the specific gene and the other genes in the module. (xlsx, 82 KB)
- File S5 - Diet specific pairwise transcript-by-phenotype correlation. Variable = Flybase transcript ID, With = phenotype, diet = diet, Pearson\_Correlation =correlation coefficient, Nobs = sample size, NegLog10\_p = negative lobe probability for a significant correlation, NegLog10\_p FDR Adjusted = False Discovery Rate, Gene ID = Flybase gene ID. (.txt, 14, 229 KB)
- File S6 - Diet specific pairwise transcript-by-phenotype correlation. Metabolite = study specific unique ID, Trait = gross phenotype, Diet = diet, Correlation = Pearson Correlation, NLP = negative log probability. (.xlsx, 48 KB)
- File S7 - Metabolite Identities. Target = study specific unique ID, best nist hit = highest spectral/retention time NIST database match, Likely Category = general chemical class of compound, Likely ID = parent compound for best NIST match, confirmed ID = chemical standard confirms ID, RT = retention time, RI = retention index. (.txt, 19 KB)
- File S8 - Correlations between expression array and Q-RT-PCR and triglyceride levels. Gene Name = Flybase Gene ID, transcript ID = Flybase transcript ID, array correlation on xxxx diet = correlation between triglycerides and gene expression on diet xxxx, NLP (array, xxxx) = negative log p-value for array by triglyceride correlation on diet xxxx, qpcr correlation xxxx diet = correlation between triglycerides and gene expression in QPCR analysis, NLP (qpcr, xxxx) = negative log p-value for Q-RT-PCR by triglyceride correlation on diet xxxx. (.txt, 5 KB)
