# Supplementary material for: Metabolomic and Gene Expression Profiles Exhibit Modular Genetic and Dietary Structure Linking Metabolic Syndrome Phenotypes in Drosophila
Source: G3 (Bethesda). 2015 Nov 3;5(12):2817–29. doi: 10.1534/g3.115.023564 (PMC4683653; doi:10.1534/g3.115.023564)
Supplement: Supporting Information [file supp_g3.115.023564_FigureS1.pdf]

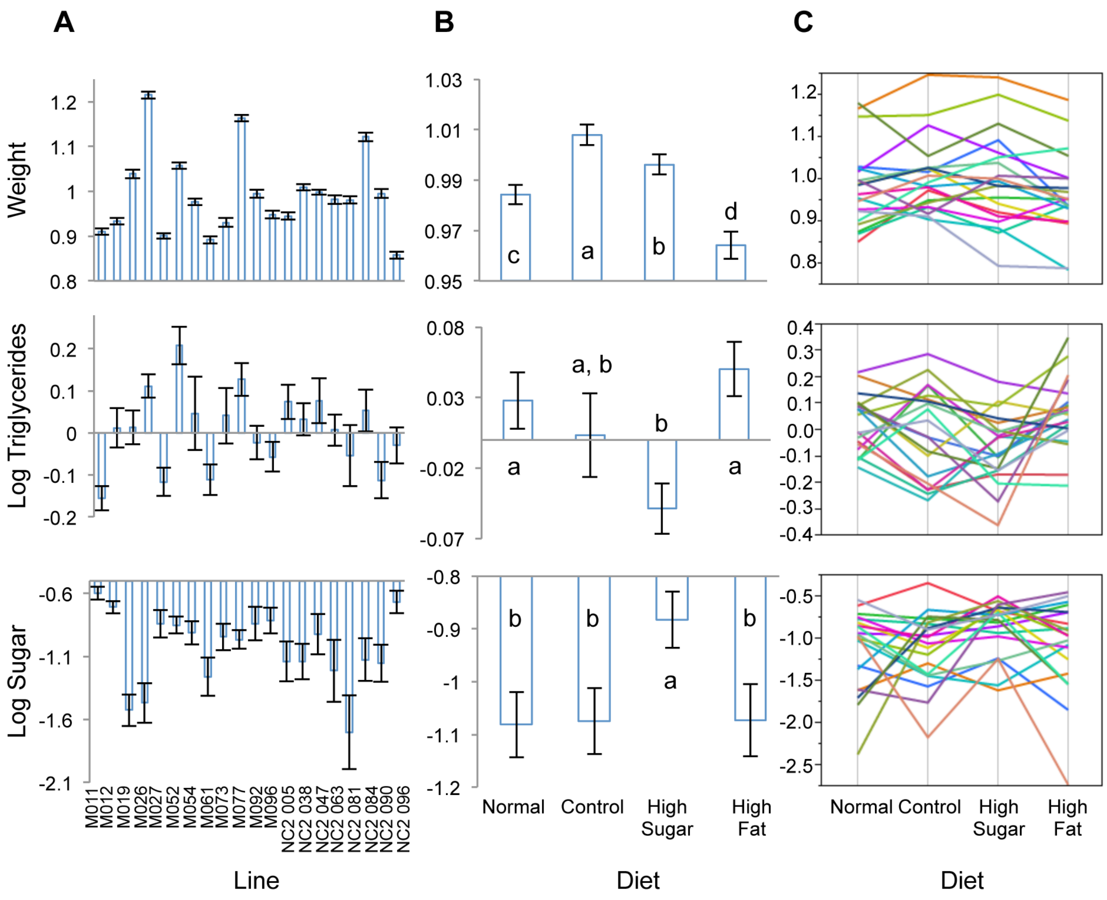

Figure S1. Genetic, dietary, and GxD interaction effects for weight, triglycerides, and sugar. Error bars are 1SE, weight is in mg, triglyceride and sugar (trehalose) is log transformed % concentration in a 3rd instar larval homogenate. A. Significant genetic variation across 20 inbred isofemale lines. B. Significant variation with diet across the four tested diets. Letters indicate statistical differences. C. Significant genotype-by-diet interaction effects with each colored line corresponding to a specific genetic line across the panels (as described in legend for Figure 3).
