# Supplementary material for: Metabolomic and Gene Expression Profiles Exhibit Modular Genetic and Dietary Structure Linking Metabolic Syndrome Phenotypes in Drosophila
Source: G3 (Bethesda). 2015 Nov 3;5(12):2817–29. doi: 10.1534/g3.115.023564 (PMC4683653; doi:10.1534/g3.115.023564)
Supplement: Supporting Information [file supp_g3.115.023564_TableS1.pdf]

Table S1. GO analysis for significant ANOVA transcripts

ANOVA Term	Number of Transcripts Significant at FDR 0.05	Top 3 GO Functional Categories (# of genes in cluster, enrichment score) Genetic effect is only the 500 most significant genes	Kegg
Genetic	9908	Metal ion binding/Oxidation reduction (122, 9.47)<br>Metabolism of xenobiotics by cytochrome P450 (23, 4.68)<br>Hydrolysis/peptidase (79, 4.53)	Limonene and pinene degradation<br>Metabolism of xenobiotics by cytochrome P450<br>Drug metabolism<br>Glutathione metabolism<br>Retinol metabolism
Diet	311	Phospholipase activity/lipase activity (14, 2.94)<br>CHK/choline kinase like (7, 2.65)<br>Oxidation reduction (35, 2.59)	Glycerophospholipid metabolism<br>Limonene and pinene degradation<br>alpha-Linolenic acid metabolism<br>Tyrosine metabolism<br>Biosynthesis of unsaturated fatty acids
Genetic* Diet	40	Puparial adhesion/salivary gland (8, 4.45)<br>Phagocytosis,engulfment (4, 1.53)	none
