# Supplementary material for: Metabolomic and Gene Expression Profiles Exhibit Modular Genetic and Dietary Structure Linking Metabolic Syndrome Phenotypes in Drosophila
Source: G3 (Bethesda). 2015 Nov 3;5(12):2817–29. doi: 10.1534/g3.115.023564 (PMC4683653; doi:10.1534/g3.115.023564)
Supplement: Supporting Information [file supp_g3.115.023564_TableS2.pdf]

Table S2. Distribution of metabolite categories with significant genetic, dietary, and genotype*diet effects

		Metabolite Category									
		amino acid	amine	saturated fatty acid	unsaturated fatty acid	steroid	monosaccharide	disaccharide	nucleic acid	carboxylic acid	sugar alcohol
	total detected	32	5	11	15	3	16	16	2	7	4
number of significant metabolites by ANOVA term (p<0.01)	Genetic	21	3	8	3	2	5	10	2	3	3
	Diet	7	1	5	3	1	4	9	1	1	3
	Genetic*Diet	2	0	3	1	0	0	0	0	0	0
