# Supplementary material for: Metabolomic and Gene Expression Profiles Exhibit Modular Genetic and Dietary Structure Linking Metabolic Syndrome Phenotypes in Drosophila
Source: G3 (Bethesda). 2015 Nov 3;5(12):2817–29. doi: 10.1534/g3.115.023564 (PMC4683653; doi:10.1534/g3.115.023564)
Supplement: Supporting Information [file supp_g3.115.023564_TableS3.pdf]

Table S3. Genes with a significant GxD interaction effect (FDR 0.05)

Gene ID	Transcript ID	Module (Average Degree)	Degree
Sgs8	FBtr0076160	1 (0.76144)	0.83629
Sgs5	FBtr0083461	1 (0.76144)	0.81872
Sgs7	FBtr0076095	1 (0.76144)	0.81845
CG7587	FBtr0083460	1 (0.76144)	0.79294
Sgs3	FBtr0076096	1 (0.76144)	0.77958
CG15404	FBtr0077578	1 (0.76144)	0.7785
CG12715	FBtr0073750	1 (0.76144)	0.76823
Eig71Ee	FBtr0075585	1 (0.76144)	0.70617
sens	FBtr0075862	1 (0.76144)	0.55409
GRHR	FBtr0079308	2 (0.55761)	0.66928
GRHR	FBtr0079310	2 (0.55761)	0.64346
Acox57D-p	FBtr0071606	2 (0.55761)	0.36008
CG15865	FBtr0074375	3 (0.52629)	0.61032
CG13397	FBtr0079711	3 (0.52629)	0.57024
CG8774	FBtr0082740	3 (0.52629)	0.56986
CG3264	FBtr0071781	3 (0.52629)	0.56219
Yp2	FBtr0071424	3 (0.52629)	0.31884
CG14998	FBtr0073265	4 (0.2491)	0.36609
CG5262	FBtr0078230	4 (0.2491)	0.34808
tth	FBtr0073849	4 (0.2491)	0.34268
CG1943	FBtr0081635	4 (0.2491)	0.34192
CG2713	FBtr0070497	4 (0.2491)	0.32075
CG11178	FBtr0073804	4 (0.2491)	0.30009
CG17618	FBtr0084306	4 (0.2491)	0.29674
Pi3K68D	FBtr0076087	4 (0.2491)	0.29545
Lasp	FBtr0100145	4 (0.2491)	0.29369
CG18445	FBtr0088434	4 (0.2491)	0.29239
CG7348	FBtr0074866	4 (0.2491)	0.23131
CG4133	FBtr0078061	4 (0.2491)	0.22822
Tig	FBtr0079275	4 (0.2491)	0.22449
eIF-4E	FBtr0076490	4 (0.2491)	0.21671
CG18136	FBtr0075040	4 (0.2491)	0.21644
comt	FBtr0073754	4 (0.2491)	0.20906
CG13675	FBtr0076730	4 (0.2491)	0.20786

CG16762	FBtr0072968	4 (0.2491)	0.20217
CG10909	FBtr0082685	4 (0.2491)	0.18481
Smox	FBtr0071192	4 (0.2491)	0.18393
CG10513	FBtr0084865	4 (0.2491)	0.17068
sug	FBtr0087789	4 (0.2491)	0.14773
Hpr1	FBtr0078667	4 (0.2491)	0.108
