# Supplementary material for: Metabolomic and Gene Expression Profiles Exhibit Modular Genetic and Dietary Structure Linking Metabolic Syndrome Phenotypes in Drosophila
Source: G3 (Bethesda). 2015 Nov 3;5(12):2817–29. doi: 10.1534/g3.115.023564 (PMC4683653; doi:10.1534/g3.115.023564)
Supplement: Supporting Information [file supp_g3.115.023564_TableS4.pdf]

Table S4. GO analysis of transcripts correlated with traits

Trait	Number of Transcripts Significant at FDR 0.05	Top GO Functional Categories (# of genes in cluster, enrichment score)	Kegg Pathways
Weight	479	Puparial Adhesion (10, 2.15)	Insect Hormone Biosynthesis
		Regulation of Protein Import Into Nucleus (5, 1.73)	One Carbon Pool by Folate
		Iron ion binding (21, 1.7)	
Triglyceride	35	Instar larval or pupal morphogenesis (5, 2.04)	Inositol phosphate metabolism<br>Phosphatidylinositol signaling system
Sugar	82	Oxidation Reduction (17, 4.0)	no sig
