# Supplementary material for: Metabolomic and Gene Expression Profiles Exhibit Modular Genetic and Dietary Structure Linking Metabolic Syndrome Phenotypes in Drosophila
Source: G3 (Bethesda). 2015 Nov 3;5(12):2817–29. doi: 10.1534/g3.115.023564 (PMC4683653; doi:10.1534/g3.115.023564)
Supplement: Supporting Information [file supp_g3.115.023564_TableS5.pdf]

Table S5. Stepwise forward regression of transcripts correlated with gross phenotypes.

Phenotype	Significance Threshold for Testing in Regression	Parameter	Estimate	Sum of Squares	P value	R Squared
Weight	Bonferroni	FBtr0077671	0.035	0.073	0.00059	0.56
		FBtr0079527	0.024	0.046	0.00618	
		FBtr0080783	-0.010	0.054	0.00309	
		FBtr0081314	-0.020	0.050	0.00443	
		FBtr0085343	0.013	0.030	0.02760	
		FBtr0086773	0.038	0.073	0.00060	
		FBtr0091822	0.014	0.039	0.01115	
		FBtr0100534	0.021	0.040	0.01036	
		Intercept	0.987	0.000	1.00000	
Triglyceride	FDR<0.05	FBtr0072168	0.297	0.981	0.00000	0.33
		FBtr0083716	-0.073	0.243	0.01008	
		FBtr0084486	0.071	0.590	0.00008	
		FBtr0085350	0.095	0.299	0.00439	
		FBtr0087157	0.026	0.223	0.01366	
		Intercept	-0.324	0.000	1.00000	
Sugar	FDR<0.01	FBtr0076003	0.104	3.050	0.00209	0.15
		FBtr0084450	0.412	3.075	0.00201	
		Intercept	-0.793	0.000	1.00000	
