# Supplementary material for: Metabolomic and Gene Expression Profiles Exhibit Modular Genetic and Dietary Structure Linking Metabolic Syndrome Phenotypes in Drosophila
Source: G3 (Bethesda). 2015 Nov 3;5(12):2817–29. doi: 10.1534/g3.115.023564 (PMC4683653; doi:10.1534/g3.115.023564)
Supplement: Supporting Information [file supp_g3.115.023564_TableS6.pdf]

Table S6. Correlations between traits and metabolites

	Metabolite				Trait Correlations (p<0.05)		
Target	Likely Category	Likely ID	Confirmed ID	Number of Correlated Traits	Weight	Triglyceride	Sugar
Target_0176	niacin			2	-0.14		-0.16
Target_0189	nucleic acid	uracil	uracil	2	-0.18	-0.14	
Target_0325	nucleic acid	pyrimidinedione		2	-0.16		0.21
Target_0349	carboxylic acid	glutaric acid		2	-0.19		0.16
Target_0426	amine	cadaverine		2	-0.21		0.27
Target_0526	catecholamine-like			2	0.21	0.20	
Target_0533	amino acid	l-dopa	l-dopa	2	0.21		-0.14
Target_0791	amino acid	endocannabinoid	arachidonoyl dopamine	2	-0.21	-0.22	
Target_0057	amino acid	alanine	alanine	1	-0.17		
Target_0074	amino acid	glycine	glycine	1		-0.20	
Target_0094	carboxylic acid			1			-0.15
Target_0125	aminomonophosphate			1	-0.17		
Target_0131	amino acid	glycine	glycine	1	-0.23		
Target_0162	amino acid	isoleucine	isoleucine	1	0.15		
Target_0164	amino acid	isoleucine	isoleucine	1	0.14		
Target_0167	amino acid	threonine	threonine	1	0.16		
Target_0178	amino acid	norleucine	norleucine	1	-0.13		
Target_0240	amide			1	-0.21		
Target_0246	amine			1	-0.18		
Target_0256	alcohol			1		0.15	
Target_0302	amino acid like			1	-0.15		
Target_0305	monosaccharide	arabinofuranose		1	-0.17		
Target_0316	amino acid	phenylalanine	phenylalanine	1			-0.15
Target_0337	amino acid	asparagine	asparagine	1		-0.18	
Target_0370	lactone			1		0.14	
Target_0374	saturated fatty acid			1	-0.13		
Target_0401	monosaccharide	fructose	fructose	1			0.16
Target_0432	monosaccharide	fructose	fructose	1		-0.13	
Target_0456	saturated fatty acid like			1		0.15	
Target_0471	sugar alcohol	myo-inositol	myo-inositol	1			-0.17

Target_0472	saturated fatty acid	Hexadecanoic acid		1	0.18		
Target_0524	sugar alcohol	scyllo-inositol		1		0.20	
Target_0532	sugar amide			1	-0.15		
Target_0579	no good hit			1			-0.15
Target_0595	saturated fatty amide	Nonamide		1			-0.19
Target_0599	unsaturated fatty acid	linoleic acid, olenic acid, or petroselinic acid	linoleic acid, olenic acid, or petroselinic acid	1	0.16		
Target_0618	saturated fatty amide	dimethyldecanamide		1	0.14		
Target_0640	aromatic secondary amine			1	0.15		
Target_0678	sugar alcohol			1	-0.26		
Target_0743	amino acid	aspartic acid-like		1			0.16
Target_0779	nucleoside	adenosine	adenosine	1	-0.18		
Target_0784	disaccharide			1	0.14		
Target_0793	disaccharide	trehalose	trehalose	1			0.14
Target_0854	alcane	Eicosane		1			-0.17
Target_0927	alcane	tridecane		1		0.15	
Target_1014	disaccharide			1	0.16		
Target_1029	ploysaccharide	pentasaccaride		1		-0.20	
Target_1154	sulfur containing	sulfur containing amino acid		1	0.20		
