# Supplementary material for: Metabolomic and Gene Expression Profiles Exhibit Modular Genetic and Dietary Structure Linking Metabolic Syndrome Phenotypes in Drosophila
Source: G3 (Bethesda). 2015 Nov 3;5(12):2817–29. doi: 10.1534/g3.115.023564 (PMC4683653; doi:10.1534/g3.115.023564)
Supplement: Supporting Information [file supp_g3.115.023564_TableS7.pdf]

Table S7. Stepwise forward regression of metabolites correlated with gross phenotypes

Phenotype	Parameter	Estimate	Sum of Squares	P value	R Squared
Weight	Intercept	1.002	0.000	1.00000	0.186
	Target_0678	-0.116	0.453	0.00000	
	Target_0472	0.021	0.085	0.00498	
Triglyceride	Intercept	0.006	0.000	1.00000	0.174
	Target_0791	-0.054	0.397	0.00373	
	Target_0524	0.046	0.254	0.01982	
	Target_0526	0.049	0.320	0.00905	
	Target_0074	-0.045	0.284	0.01389	
	Target_1029	-0.049	0.356	0.00600	
Sugar	Intercept	-1.025	0.000	1.00000	0.102
	Target_0426	0.177	5.430	0.00017	
	Target_0595	-0.135	2.438	0.01084	
