# Supplementary material for: Metabolomic and Gene Expression Profiles Exhibit Modular Genetic and Dietary Structure Linking Metabolic Syndrome Phenotypes in Drosophila
Source: G3 (Bethesda). 2015 Nov 3;5(12):2817–29. doi: 10.1534/g3.115.023564 (PMC4683653; doi:10.1534/g3.115.023564)
Supplement: Supporting Information [file supp_g3.115.023564_TableS8.pdf]

Table S8. GO analysis of genes overlapping traits

Trait1	Trait2	# of shared transcripts (p-value threshold)	GO Functional Term (# of genes in category, p-value)	KEGG Pathway
Weight	Sugar	147 (0.01)	Oxidation Reduction (19, 2.3E-5)	Valine, leucine and isoleucine degradation (4, 7.6E-3)
			branched chain family amino acid metabolic process (3, 1.9E-3)	
Weight	Triglyceride	66 (0.01)	protein homooligomerization (2, 1.6E-2)	none
Triglyceride	Sugar	93 (0.05)	neuropeptide signaling pathway (3, 1.5E-2)	none
			regulation of transcription (10, 1.9E-2)	
			structural constituent of chitin-based larval cuticle (3, 2.3E-2)	
