# Supplementary material for: Metabolomic and Gene Expression Profiles Exhibit Modular Genetic and Dietary Structure Linking Metabolic Syndrome Phenotypes in Drosophila
Source: G3 (Bethesda). 2015 Nov 3;5(12):2817–29. doi: 10.1534/g3.115.023564 (PMC4683653; doi:10.1534/g3.115.023564)
Supplement: Supporting Information [file supp_g3.115.023564_TableS9.pdf]

Table S9. GO category enrichment by diet for weight, triglyceride, and sugar

Trait	Diet	Top GO Category	Genes in Category	p-value
Weight	Normal	transition metal ion binding	13	1.30E-02
	High Fat	DNA binding	48	9.40E-04
	Control	nucleosome organization	8	1.90E-06
	High Sugar	nucleosome organization	8	3.20E-04
Triglyceride	Normal	ubiquitin-protein ligase activity	2	4.10E-02
	High Fat	purine ribonucleotide binding	12	1.10E-04
	Control	DNA binding/transcription factor	22	3.70E-03
	High Sugar	hemopoisis	3	1.10E-02
Sugar	Normal	oxidation reduction	9	1.70E-03
	High Fat	chromoprotein	2	1.80E-02
	Control	cytoskeletal protein binding	9	5.80E-05
	High Sugar	electron transport chain	3	3.00E-02
