# Supplementary material for: Metabolomic and Gene Expression Profiles Exhibit Modular Genetic and Dietary Structure Linking Metabolic Syndrome Phenotypes in Drosophila
Source: G3 (Bethesda). 2015 Nov 3;5(12):2817–29. doi: 10.1534/g3.115.023564 (PMC4683653; doi:10.1534/g3.115.023564)
Supplement: Supporting Information [file supp_g3.115.023564_TableS10.pdf]

Table S10. Gene IDs for transcripts correlated with traits across multiple diets

Trait	Diets	Transcript ID	Gene ID	Gene Function
Weight	4	FBtr0079527	CG7106	galactose binding
		FBtr0083603	Transcriptional adapter 2A; DNA-directed RNA polymerase II 16 kDa polypeptide	DNA binding/mRNA transcription
		FBtr0085650	prolyl-4-hydroxylase-alpha EFB	peptidyl-proline modification
Triglyceride	2 or more	FBtr0072168	CG3173	unknown
		FBtr0077023	Cuticular protein 65Aw	structural constituent of cuticle
		FBtr0079549	CG7219	endopeptidase inhibitor activity
		FBtr0084063	modifier of mdq4	chromatin organization
Sugar	2 or more	FBtr0079387	CG11322	unknown
