# Supplementary material for: Metabolomic and Gene Expression Profiles Exhibit Modular Genetic and Dietary Structure Linking Metabolic Syndrome Phenotypes in Drosophila
Source: G3 (Bethesda). 2015 Nov 3;5(12):2817–29. doi: 10.1534/g3.115.023564 (PMC4683653; doi:10.1534/g3.115.023564)
Supplement: Supporting Information [file supp_g3.115.023564_TableS11.pdf]

Table S11. GO category enrichment for genes correlated with weight on multiple diets

Trait	Number of Diets with Significance	Distinct GO Category	Genes in Category	p-value
Weight	2 or more	nucleosome organization	6	1.40E-05
		proteolysis	11	3.40E-02
Weight	3 or more	regulation of histone modification	2	1.20E-02
		peptidyl-proline modification	2	1.50E-02
