# Supplementary material for: Metabolomic and Gene Expression Profiles Exhibit Modular Genetic and Dietary Structure Linking Metabolic Syndrome Phenotypes in Drosophila
Source: G3 (Bethesda). 2015 Nov 3;5(12):2817–29. doi: 10.1534/g3.115.023564 (PMC4683653; doi:10.1534/g3.115.023564)
Supplement: Supporting Information [file supp_g3.115.023564_TableS12.pdf]

Table S12. Primers used in Fluidigm Q-PCR analysis

Gene	Forward	Reverse
Acox57D-p	GCGAGGAAGTGCTAAAGTTCAA	GCAGCATCTCCGACAAATCC
Act5C	ATGGGCCAGAAGGACTCGTA	CGTGCTCAATGGGGTACTTCA
Amyrel	TGGATGCCGCCAAGCATA	GGACGGGAGTTGTGAGGAAA
CG11317	ATGGCGGATGACGATGATGAC	AACTGGCGGTGACAATGGAA
CG12448	TCCACAGAATGCGAAAGGAAC	AAGCGGTAGCCAAAGGTACTA
CG12824	GCGATCGCCTACCTCATCATA	CTGCATGGACGCTATGTGAAC
CG13609	ATCTACTCGCGCGATCATCA	TCCTGCAGTCGTTCGCTAA
CG13947	GTGGGGACTGCCTCCAAA	TGGATTCGGTGGTCGTTGAA
CG14805	GACGGCAAGGTGTTTGTCTTTA	CCACAAAGGCCTGGATTTCC
CG15111	TCGGTATTCCGTAACGTTCCA	TTCCGGCTTGGTGAGATCAA
CG15483	TTGGTGCCCGACTATATGCA	CACATGGACCAACGGTGAA
CG15820	CGGTGGAACAAACTACAAGGAA	CAACGGCCTGCTATGTGAC
CG15865	GCCAGGAACCTGCTCTTCAA	CACGTCAACGTTCTCAAAGACC
CG17134	TCTTCGGCATCCAGCACTTA	ACAGGAAACCCGACAGACTAC
CG17322	CCCTCCACCTTGACAGACAC	GGTATTGCAGTCCTCTCTTCCC
CG17806	GAGCAACATCCTCAAGCTGAC	TCCAGCAAGCAGGATAAGCA
CG18477	TTTACGGGCTGGGGAAAGAA	TTGGACCACCGGCAATGATA
CG18563	CTAGCGGACCGAATGAGGAA	AGACTGTTGGTCTCGTTGGAA
CG2269	TCTGCGGTGTTCACCAGATA	AGTACTGATAGTCGCAGGCTAC
CG32212	TCTGGCTTACTCTGCTCCTCTA	GATACTGGTGGCGGTCACA
CG32335	GCCCCTCTCGGAACTGAAA	TAAATCCGGGCAAGCTTCCA
CG34104	AACGGAGGCCGCAACAA	CCAGGACGCACTTGATCGAA
CG3558	ACCCAAAAGCGGAACCACTA	ATGCACGTACGGGATAAGCA
CG4891	ATGGCGGAGGTCATAAGAACA	TCCTGCCACTGATGCCTTTA
CG5130	CTCGGCGGTTGTATCATCAC	AGCTACTCCGGAGCCATTTA
CG5131	TCTTGAGCGCCATGTTCCA	AGCTAGGGCTTTGGACTTGAC
CG5612	GCCAATCACTTGCGTGGAAA	ACGATGAGGCGATTCACCAA
CG7634	GCACTGCGTAGTATTCGGTAC	CTCCCATAACTTCCTCCACGTA

CG8311	ACGGATGTCTTCAGCCTCA	AGCGGCAGCAGTAGACATA
CG8858	GAAACTGGACCGAATGCAGAA	CATCCGAGTCCGCATTTCC
CG9139	CTCGATGTGCTTCCGAGAAAA	CGAGGAAGAGGATCCAGGAC
CHKov1	CGAAAACTTTGTTGGCGATTCC	CGATAGCGGGGATTGTTGTAC
Cp110	ACTTGGACGGGCAGCCTATA	TACTGGCCATGTCGGATCCC
Cyp6d2	ATCCCCATCGCTTCGATTCA	CACCTTGGAGTTGACTTTTCCC
Fs	CCCAACTGGAGGTCGCCTA	ACGCAGGTCAATCCGTTCAA
g	CGCGGCATTAGGAACAACAA	CAGCTCCTGTTTGATCTCCTCA
Gapdh1	AGGCTGGCATTTCGCTGAA	ATGACGCGGTTGGAGTAACC
GstD9	GCCACCGTTTCCACTTTTGAA	TTGGCATTGTCGTACCACCTAA
Idgf5	CGGTGGCCAACTGCTAAC	GGTTGACGAAGTCCACGTTAC
Lcp9	ACGAGGAGGCTGATGTTGTA	GGCGCGAATATGGTTGGATA
lola	GTCGTCGCCGAAAAGTCC	CTGTCTGCTGGAGGATTGAC
lsn	GTGCACCAGGAGATCACCAA	TTGCCCAGCTTGTGAACCA
mAcR-60C	GACTCCAGCAAGAGATCAAACA	CAGGTGTCATGGTCGTTTCC
mthl9	TCAAATCCTGGTGGGCCATTA	CGAACTTGTATACGCGAGCATTA
Picot	TCTGGAGAGGCAGAGGAGTA	CTCGCCATCCTGAGAGTCA
SP555	GAGGGCACACTCACCTTCTA	AGAGCATACGATGGGGTACA
spel1	AGCTGCAGGAGCATGTCAA	CCATGTTGGGTCCCGTGATTA
Yp2	GCCCAACCTGAAGGAGGTTA	GGGCTTGAAGACATGGTTGAA
