# Supplementary material for: Metabolomic and Gene Expression Profiles Exhibit Modular Genetic and Dietary Structure Linking Metabolic Syndrome Phenotypes in Drosophila
Source: G3 (Bethesda). 2015 Nov 3;5(12):2817–29. doi: 10.1534/g3.115.023564 (PMC4683653; doi:10.1534/g3.115.023564)
Supplement: Supporting Information [file supp_g3.115.023564_FigureS2.pdf]

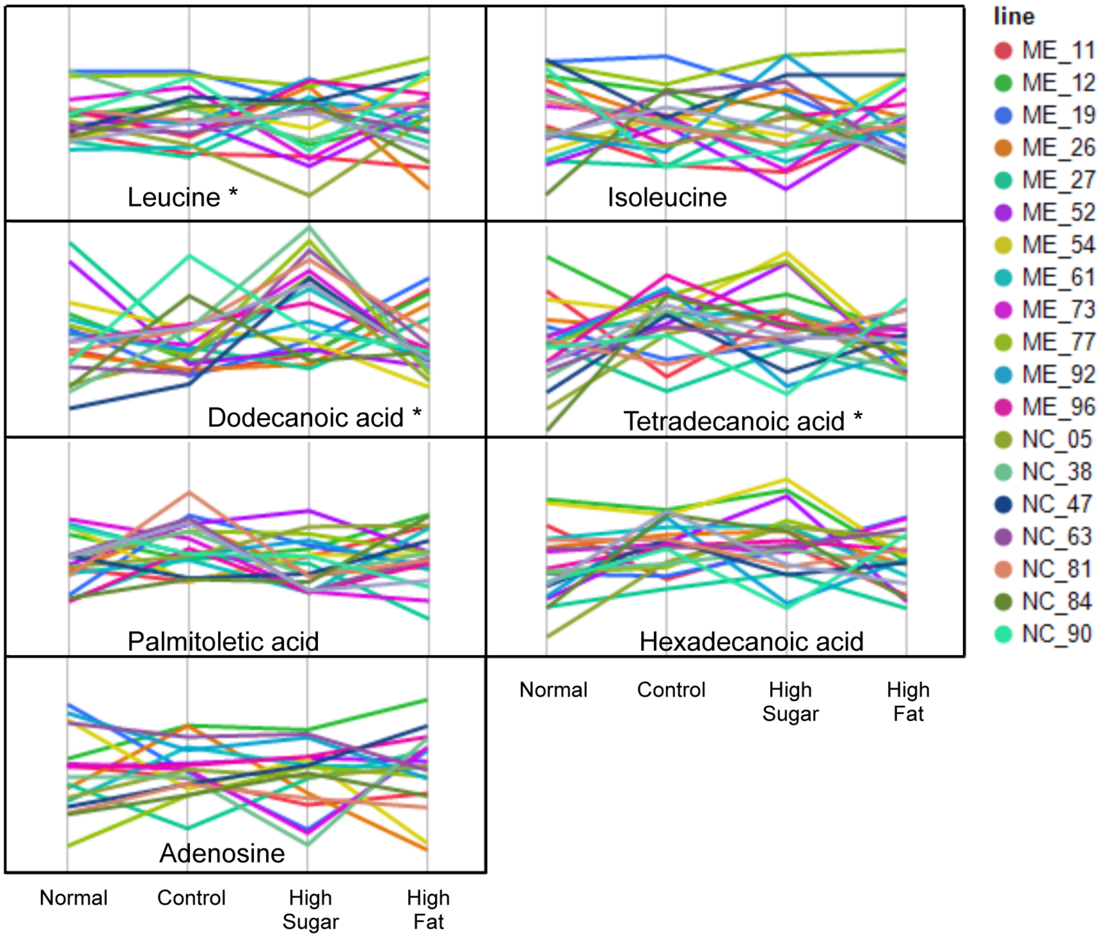

Figure S2. Metabolites with significant genotype-by-diet interaction effect and confirmed identities. Significance determined at $p < 0.01$, * indicates Bonferroni significance. Significant metabolites include branch chain amino acids (leucine and isoleucine), long chain saturated fatty acids (dodecanoic, tetradecanoic, hexadecanoic), an omega-7 mono-unsaturated fatty acid (palmitoletic), and a purine nucleoside (adenosine).
