# Supplementary material for: Metabolomic and Gene Expression Profiles Exhibit Modular Genetic and Dietary Structure Linking Metabolic Syndrome Phenotypes in Drosophila
Source: G3 (Bethesda). 2015 Nov 3;5(12):2817–29. doi: 10.1534/g3.115.023564 (PMC4683653; doi:10.1534/g3.115.023564)
Supplement: Supporting Information [file supp_g3.115.023564_FigureS3.pdf]

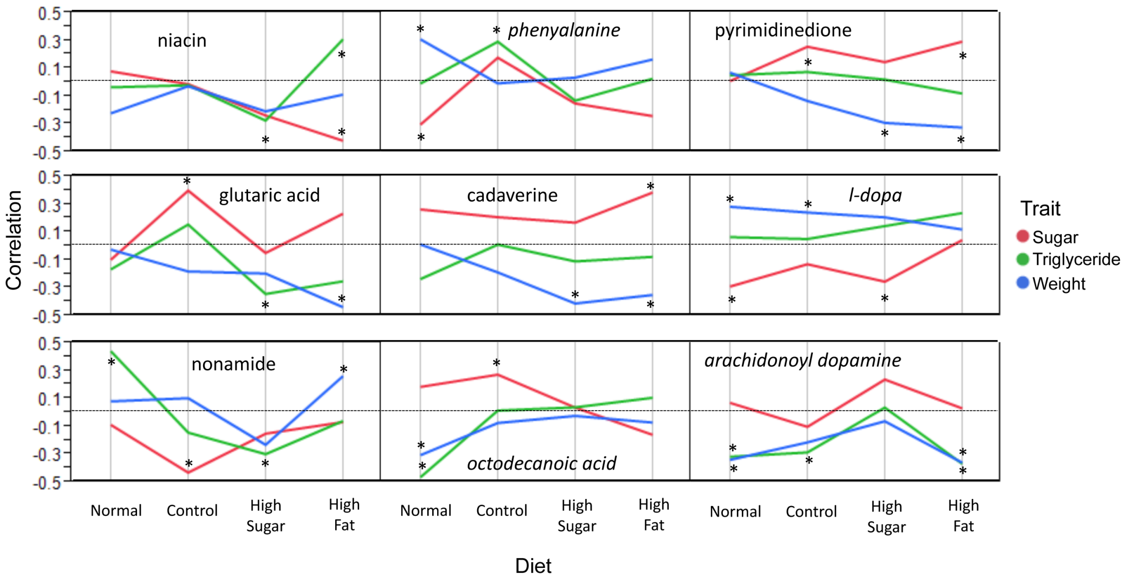

Figure S3. Metabolites showing strong correlation with weight, triglycerides, or total sugar. Names indicated in italics are experimentally confirmed identities. Values above the dashed line at 0 indicate a positive correlation, while values below the dashed line indicate a negative correlation. * indicates a significant correlation at $p < 0.01$.
